# Surgery, Fame, and Misfortune: The Life of Bronisław Kader

**DOI:** 10.1007/s00268-012-1588-y

**Published:** 2012-04-10

**Authors:** Anita Magowska

**Affiliations:** Department of History of Medical Sciences, Poznan University of Medical Sciences, ul. Przybyszewskiego 37A, 60-356 Poznan, Poland

## Abstract

Bronisław Kader (1863–1937) introduced one of the traditional methods of gastrostomy. He was a Polish doctor who had been trained by such eminent surgeons as Ernst von Bergmann, Otto E. Küstner, Jan Mikulicz, and Eduard von Wahl. The Kader method implies blunt division of the left rectus muscle and opening of the stomach by a very small incision. A drainage tube is then inserted and fastened to the stomach wall by a stitch. Next, the stomach wall is sutured to the abdominal wall in a manner that places the tube in a tunnel surrounded by serosa. In comparison to others, Kader’s method of gastrostomy was considered simpler, cheaper (fewer stitches), speedy, and safe. Although recommendations to perform gastric fistula were limited at the time, the value of gastrostomy remains undisputable. This is a method of choice for securing alimentation in cases of intractable stenosis of the pharynx or esophagus, which are usually due to cancer, chemical burns, trauma, or congenital defects. Nowadays, it is performed endoscopically or laparoscopically. This article presents the life history of Bronisław Kader, the eponymous of this method and a gifted surgeon who lost his eyesight at the height of his fame.

## Educational Background

Bronisław Kader (full name: Kadaras-Kader) was born on May 6, 1863 in Vilnius in Russian Poland. (At that time Poland was partitioned between three superpowers: Austria, Germany, and Russia.) His father, Andrzej Kadaras-Kader, was a vicar and a principal administrator of the Calvinist churches in the former Grand Duchy of Lithuania, and his mother, Anna, coming from the Lipiński family, was the owner of the Rygmuntyszki estate. Even though Calvinism was a rare denomination in a country mostly inhabited by Catholics, by no means did it interfere with his deep emotional attachment to Poland in which Bronisław was brought up [1].

In 1881, he completed his secondary school education in Vilnius and left for Dorpat (today Tartu, the capital of Estonia) to study medicine under famous German scholars and to enjoy some freedom there [2]. Kader received his diploma in medicine in 1889. This delay was because of his work in the surgical clinic run by Eduard von Wahl (1833–1890), an expert in vascular, abdominal, and gland surgery [3], and in the clinic for women run by Otto E. Küstner (1849–1931), who specialized in the use of forceps to assist delivery [4].

After graduation from Dorpat University, Kader took up the position of assistant in Wahl’s clinic. In the doctoral dissertation entitled “The case study of topical flatulence resulting from intestinal obstruction” (“Ein experimenteller Beitrag zur Frage des lokalen Meteorismus bei Darmocclusion”), guided by Wahl as a supervisor and based on experiments carried out on more than 100 animals (dogs, cats, pigs, sheep, rabbits, horses), he gave a detailed explanation on the anatomopathologic changes in various cases of intestinal obstruction [5]. In 1891, after Wahl’s death, Kader defended his thesis and soon gave members of the German Association of Surgeons an account of it, making such a good impression that he was immediately admitted to the prestigious organization. He then moved to Berlin and became an assistant in the surgical clinic run by the famous Ernst von Bergmann (1836–1907), the inventor of heat sterilization of instruments and dressings. However, when Jan Mikulicz (1850–1905), a worldwide renowned surgeon, offered him the position of assistant in a surgical clinic in Breslau (now Wrocław), Kader accepted this proposal willingly as it was a great chance to be trained by the Polish surgeon.

The work in Mikulicz’s clinic was hard and demanding because all of the members of the research staff were subjected to a hierarchical, military-like regimen. In the clinic, doctors were allowed to discuss only professional matters, and addressing each other as “colleague” was strictly forbidden. With regard to tradition, surgery was considered a true vocation; hence, doctors working in the clinic could not be married, although Mikulicz had a family. Nevertheless, this was an excellent opportunity to gain comprehensive surgical training. On the one hand, Mikulicz required doctors to perform surgical operations precisely and safely for patients; on the other, he made his research staff learn pathomorphology, internal medicine, and bacteriology because such knowledge was needed to make a quick, accurate diagnosis without consultation [6].

In Breslau, Kader developed his outstanding surgical techniques and published a few important works. His valuable publications in the field of orthopedic surgery, including “Langjährige Neuralgie des rechten plexus cervicalis und brachialis in Folge von narbiger Verkürzung des linken Kopfnickers: Vollständige Heilung nach Tenotomie dieses Muskels” (Multiannual neuralgia of the right cervical and brachial plexus resulting from cicatricial shortening of left side muscle groups responsible for head movement: a complete recovery due to the tenotomy of these muscles), which contributed to his admission to the German Association of Orthopedists and Urologists [7].

In 1895, he was promoted to the position of Mikulicz’s deputy and received official permission to practice as a doctor in Germany—then a rare privilege for Poles. Two years later, he wrote a thesis entitled ”Klinische Beiträge zur Aetiologie und Pathologie der sogenannten primären Muskelentzündungen” (A case study for the etiology and pathology of so called primary muscle inflammation) and earned his habilitation, the highest academic qualification in Germany entitled to the position of *Privatdozent*, equivalent to associate professor in English-speaking countries. The thesis was published in the journal *Mitteilungen aus den Grenzgebieten der Medizin und Chirurgie*, which had been founded by Mikulicz to develop interdisciplinary research in both medicine and surgery [8].

After gaining his habilitation, Kader took a sabbatical to visit most of the most significant surgical clinics in Austria, Germany, Switzerland, and Scandinavian countries. After returning, he received offers to take up a professorship in surgical clinics in Basel, Kharkov, and Halle. He neither accepted them nor agreed to hold a post of the executive manager of the Red Cross Hospital in Łódź, then a Polish city under Russian occupation. However, with Mikulicz’s permission, he commuted to Łódź from Breslau to supervise the hospital that was under construction. When the building work was completed, Kader filled all the positions of consultants and specialists with Polish doctors, and he took all the necessary steps to ensure that office documents were written in the Polish language, which was unprecedented in Russia; but in the end he gave up the work. At the time, his one and only hope was to get an offer from the University in Cracow, then a Polish city that was under Austrian occupation [9].

## Surgical Clinic in Cracow

**Fig. 1:**
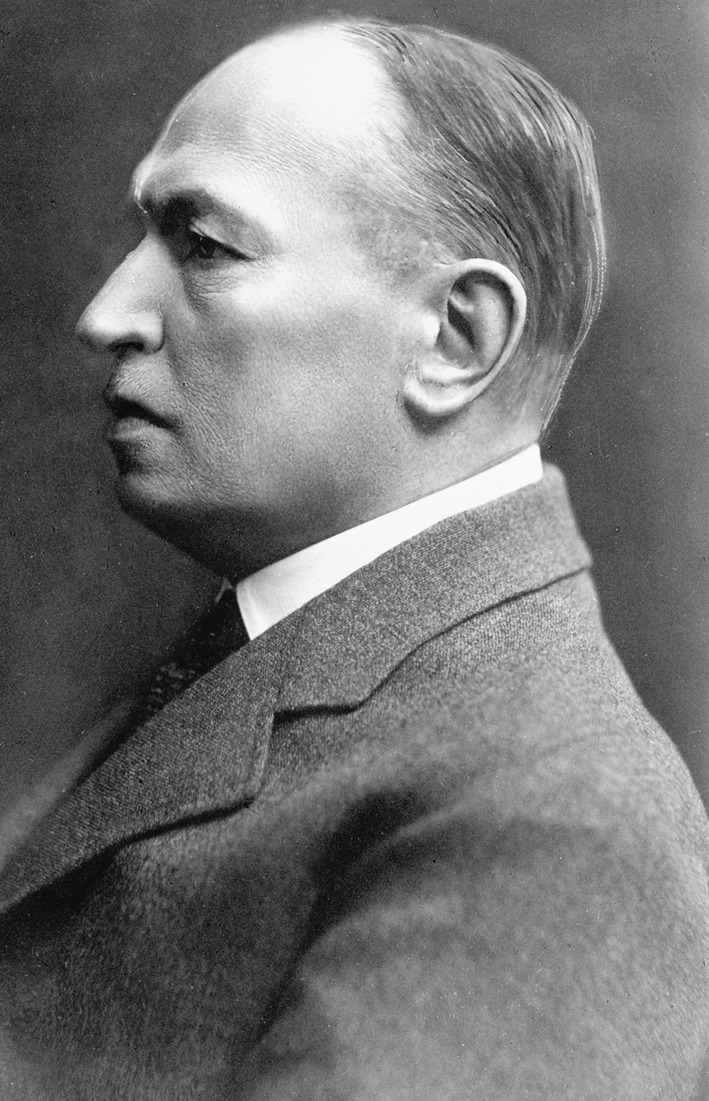
Bronisław Kader in 1899. (Courtesy of the Polish National Digital Archives)

His hopes were fulfilled in 1899, when the Austrian Ministry of Science appointed him to the chair and clinic of surgery at Cracow University (Fig. 1). After arriving in Cracow, he found that the working conditions were very bad and much worse than he ever could of imagined. The clinic building was too small, its rooms were cramped, and medical instruments and equipment were scarce—not to mention the fact that an X-ray device was missing. Because of finances provided by the Austrian authorities were insufficient, the number of admissions had to be limited to no more than half of the 42 beds available in the clinic. Moreover, patients were hospitalized only during the academic year; and during summer holidays, Christmas, and Easter the clinic was closed. Therefore, inhabitants of Cracow consciously reported sick at St. Lazarus Hospital, which was situated nearby on the other side of the street and offered its patients a large surgical ward equipped with 300 beds. Only those patients turned away from St. Lazarus Hospital or those who wanted to be operated on by the professor came to the clinic [1].

Kader thus had reasons to make an effort to rebuild and modernize the clinic. Because of the lack of financial support, he used his own money to organize and equip the clinic with devices and to create an aseptic operating room, a sterilization room, an X-ray laboratory, an orthopedic ward, a factory for making prostheses, and two schools for the disabled (craftsman and agricultural). Furthermore, he paid salaries to five members of his personnel, still using his own financial resources, which were never repaid completely. To increase the number of patients for didactic purposes and research, Kader tried to combine the clinic with the surgical ward of St. Lazarus Hospital; facing the disapproval of local doctors, however, his attempts failed.

Following Mikulicz’s school, Kader demanded a lot from his coworkers, training them in a strict but respectful manner. A few of his pupils became heads of surgical chairs and clinics, including Vaclav Chlumsky in Bratislava (former Czechoslovakia), Kornel Michejda (1887–1960) in Vilnius, and Zygmunt Radliński (1874–1941) in Warsaw. Others were in charge of hospital wards in Poland, and one migrated to the United States and worked as a consultant [10].

Apart from his clinical practice, Kader contributed to the modernization of medicine in Poland. At medical association meetings, he demonstrated how to simplify incisions, increase the safety of surgical procedures, and shorten the period of hospitalization [11]. He taught how to perform an aseptic operation with sterilized sutures and rubber plasters [12]. He gave lectures on intravenous and spinal anesthesia by the Corning-Bier method [13]. He also presented some of Mikulicz’s techniques for abdomen, thyroid, bladder, and joint surgery [1].

When World War I broke out, the Austrian authorities turned the clinic into a military hospital. Kader was forcibly conscripted into the Austrian army as a senior doctor at the rank of colonel. He was responsible for inspecting the surgical wards in all of the military hospitals in the Cracow fortress organized by the Austrians. Treating the victims of war was a challenge for Polish doctors. They were not accustomed to providing surgical procedures and treatment for hundreds of injured soldiers. Hence, Kader organized systematic training on medical topics that were essential during wartime. He himself went to Lvov, Vienna, and Berlin to participate in conferences and other meetings for military surgeons.

When the front line moved eastward, far from Cracow, Kader tried to get himself dismissed from the Austrian army so he could continue his scientific career, but he failed. Instead he was ordered to carry out inspections in front-line hospitals. A dramatic change in his life happened in 1917 while operating on a wounded soldier. He was accidentally cut by an assisting doctor, which resulted in sepsis. He spent a year and a half in the hospital in very poor condition, and he experienced the first signs of the deterioration of his eyesight. As diagnosed later, this ailment was caused by a pituitary gland tumor. He was sent for surgical treatment to Berlin, but when he eventually went there the revolution broke out and the city was plunged into bloody riots. He had to return to Cracow [1].

When he arrived home, World War I had just finished, and Poland had regained its independence. Kader could have been happy if not was not for suffering so much. He urgently needed a neurosurgical procedure, but in Europe only two surgeons, both from Mikulicz’s school, were capable of performing the operation. One was Gustav Killian (1860–1921) from Berlin, and the other was Anton Eiselberg (1860–1939) from Vienna [14]. Unfortunately, Killian was suffering from stomach cancer, and he did not feel well enough to undertake any further operations. Eiselberg, Kader’s friend from his time in Breslau, remained the only one who could help him. Nevertheless, when Kader arrived in Vienna in November 1920, Eiselberg doubted about his chances of surviving such a procedure and finally refused. Instead, he recommended palliative treatment in the form of x-ray deep irradiation which resulted in Kader suffering from severe anemia and deterioration in his general well-being, so the therapy had to be discontinued. A few months later, Oscar Hirsch from Vienna undertook the surgery, but he did not reach the tumor transnasally. Eventually, in 1922 Eiselberg decided to use his own operative method, but he also failed to reach the tumor. What is worse, the operation led to severe postoperative wound inflammation. Once again Kader’s life was in jeopardy, and he struggled to survive [10].

**Fig. 2:**
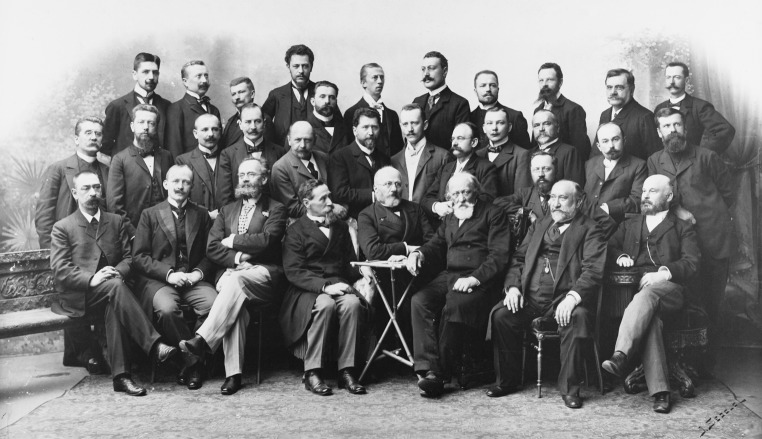
Bronisław Kader (*the man wearing glasses in the middle*) and the staff of the surgical clinic in the Cracow University in 1928. On his *right side* is Maksymilian Rutkowski, his follower. (Courtesy of the Polish National Digital Archives)

In 1923, Kader returned to Cracow but his disability had changed him into a frail and depressed elderly man. The tumor deprived him of eyesight in his left eye and caused optic nerve atrophy in his right eye. Blindness for such an energetic and active man was a personal tragedy. At the request of the Medical Faculty of the Jagiellonian University, Kader was dismissed from managing the surgical clinic. Despite this, he still held the position of a full professor and was involved in lectures on theoretical surgery. Thanks to his wife Charlotte Joanna (maiden name Teucher) who was born in Dresden and whom he married in 1923 shortly before his 60th birthday (according to the old tradition mentioned above, surgeons preferred to live alone), he was able to introduce new materials into his lectures. Charlotte Joanna read him the latest articles published in German and Polish surgical journals. The responsibilities of the head of the clinic were taken over by Maksymilian Rutkowski (1867–1947), who had previously been the head of the surgery ward in St. Lazarus Hospital. Rutkowski was also recommended for a surgery professorship, but acting on a noble impulse he did not accept this offer until Kader’s retirement in 1928 (Fig. 2).

For his merits in the field of surgery, Kader was awarded honorary membership by the Polish Surgeon’s Association, Cracow Medical Association, and the Vilnius Medical Association [10]. Kader died on October 24, 1937 in Cracow. He was buried in the Evangelical Cemetery, Vilnius, which was closed World War II [15].

## Kader’s Surgical Techniques

First described and explained in 1896, Kader’s method of gastrostomy became a method of choice for many surgeons for almost 100 years [16]. It is worth recalling that the first gastric fistula to feed a patient was performed for esophageal obstruction by the French surgeon Sédillot in 1849 [17]. From this time, surgeons criticized gastrostomy because it did not provide a full recovery. At the end of the 19th century, the approach to the procedure changed completely, and a few methods of gastrostomy were presented in the medical press. Those used most often were introduced by Witzel (1891), Stamm (1894), Janeway (1913), and Hirsch (1915) [18]. The advantages of Kader’s method over the others was the possibility of opening the stomach lumen by a very small incision in its front wall and make a tunnel lined with serosa, which creates favorable conditions for self-closure of the gastrostomy after drainage-tube removal [19]. As such eminent surgeons as Jan Mikulicz and Berkeley Moynihan emphasized, Kader’s technique is simple, speedy, easy, and economical [20]. Nowadays, this method has its own adherents as well because it is the only gastrostomy method in which the results were assessed in so many patients and over such a long period of time; furthermore, cost and complication rates are low [21].

The first percutaneous endoscopic gastrostomy was performed in 1980, and in 1991 surgeons conducted the first laparoscopic gastrostomy. These advances in surgical technology decreased interest in Kader’s classic procedure but did not render it obsolete. The number of inserted stomach fistulas is decreasing, but the procedure is still performed laparoscopically and endoscopically for complications resulting from digestive system disorders, critical conditions caused by extensive surgery [17], head and neck tumors [22], and on elderly patients [23].

It is worth mentioning that the excellent practical outcome of Kader’s method of gastrostomy in controlling the opening into the stomach encouraged Gibson to adapt this procedure for operations on the urinary bladder. Described about 100 years ago, Gibson’s technique is carried out by making a small incision in the urinary bladder wall, inserting a rubber tube, and suturing it to the bladder wall by means of a Lembert suture [24]. Gibson also recommended the application of Kader’s gastrostomy method to making a valvular opening into the cecum for treating various forms of chronic colitis by irrigation [25].

Bronisław Kader is also eponymous of Wahl-Kader’s syndrome, which is a characteristic of intestinal obstruction resulting from bowel infarction when it is possible to diagnose topical intestinal bulge corresponding to a prominent intestinal loop deprived of peristalsis [4].

Even though the remaining surgical techniques introduced by Kader have not stood the test of time, it is worth recalling them as they prove that Kader was an evident link between Mikulicz’s school and Central European surgery, especially in Poland. Using his own gastroenterostomy method in cases of stomach cancer, where he combined the stomach with an intestine in accordance with the vicious circle rule (*circulus vitiosus* was the term introduced by Mikulicz), thereby reducing the need for enteroanastomosis, jejunostomy, or gastrostomy [26]. The surgical method of treating torticollis developed by Mikulicz and Kader was considered classic [27, 28].

The Great War and eyesight loss troubled Bronisław Kader when he wanted to describe all of his innovative operative techniques in surgical journals. These techniques, presented during medical association meetings, are as follows: tendoplasty performed on patients who underwent celebral palsy [29]; transplantation of hip joints from cadavers [1]; removal of an osteoma from the ear canal [30]; and goiter operations. Kader demonstrated these methods in Cracow, and some of them were used in Poland during the following years [10].
